# First principles investigation on energetics, structure, and mechanical properties of amorphous carbon films doped with B, N, and Cl

**DOI:** 10.1038/s41598-019-55488-x

**Published:** 2019-12-12

**Authors:** Hwanyeol Park, Daekwang Woo, Jong Myeong Lee, Se Jun Park, Sungwoo Lee, Ho Jun Kim, Euijoon Yoon, Gun-Do Lee

**Affiliations:** 1 Department of Materials Science and Engineering, Seoul National University, Seoul, 08826 Korea; 2 Memory Thin Film Technology Team, Samsung Electronics, 445-701 Giheung Hwaseong Complex, South Korea; 3 Department of Mechanical Engineering, Dong-A University, Busan, 49315 South Korea; 4 Research Institute of Advanced Materials and Inter-university Semiconductor Research Center, Seoul National University, Seoul, 08826 South Korea

**Keywords:** Materials science, Mathematics and computing, Physics

## Abstract

Amorphous carbon (a-C) films have received significant attention due to their reliable structures and superior mechanical, chemical and electronic properties, making them a strong candidate as a hard mask material. We investigated the energetics, structure, and electronic and mechanical properties of the B, N, and Cl doped a-C films based on density functional theory (DFT) calculation. Our DFT calculated results clearly show that introducing B and N atoms into a-C films makes the bulk modulus slightly reduced as a function of the concentration increases. Interestingly, it is noted that introducing Cl atom into a-C films makes the bulk modulus is drastically reduced, which suggests that the films softened by Cl doping would relieve residual stress of the individual layers within the overall stacks in integrated semiconductor devices. These requirements become more important and increasingly more challenging to meet as the device integrity grows. In the perspective of F blocking nature, B doping into a-C films pulls in and captures the F atom due to the strong bonding nature of B‒F bond than C-F bond. Unlike the B doping, for the N doped a-C film, F atom has extremely large diffusion barrier of 4.92 eV. This large diffusion barrier is attributed to the electrostatically repulsive force between both atoms. The Cl doped a-C film shows consistently the similar results with the N doped a-C film because both N and Cl atoms have large electro-negativity, which causes F atom to push out. If one notes the optimized designing with the suitable doped characteristics, our results could provide a new straightforward strategy to tailor the a-C films with excellent mechanical and other novel physical and chemical properties.

## Introduction

Amorphous carbon (a-C) films have received significant attention due to their reliable structures and superior mechanical, chemical and electronic properties^1–3^, making them a strong candidate as a hard mask material for the fabrication of future integrated semiconductor devices. For wider applications, however, it is still necessary to overcome the current limitations such as high internal stresses, low blocking nature of fluorine attack to sub-layers, and low temperature stability^4^. In order to eliminate the drawbacks and enhance properties of the a-C films, tremendous approaches have been carried out. P.K. Chu *et al*.^5^ found that the most common chemical bonds in a-C are sp^2^ and sp^3^ hybridized bonds and a high percentage of sp^3^ bonds than the sp^2^ bonds is favored in the a-C films. So far, there have several reports^6–8^ on doping of a-C films with various elements such as Si, Al Cr, Ti, and W in order to optimize the materials properties. These approaches substantially present the deep insight of mechanisms for enhancing many properties (total energy, internal stress, electronic structure, optical gap, and diffusion of interstitial atoms). R. Glmore *et al*.^6^ have reported that in contrast with hydrogenated a-C films, Si doped a-C films have low friction after the creation of SiO_2_ particles from a chemical reaction and improve the behavior of high temperature. K. Trojan *et al*.^7^ have stated that metals doped a-C films, such as Ti and Al, significantly reduce surface energy. D. -Y. Wang *et al*.^8^ have proved that the dopant elements of metals reduce internal stress and activity of the chemical reaction between additives and a-C films by forming metal oxide covalent bonds, which localize the electrons of the metal.

Even though the several experiments on the doping of a-C films have been reported, no guidelines exist for enhancing materials properties of a-C films as an etch hard mask since the relation between structural properties of a-C film and its etching characteristics remains unclear. First principles calculations would provide a powerful tool to figure out structural and electronic details and capture a deeper understanding of the stress reduction mechanism of elements doped a-C films^9,10^.

The reason why we have chosen those three main dopant elements is because a-C films doped with B, N, and Cl elements are mainly used in semiconductor devices rather than other dopants such as Si, Cu, Cr, Ti, W, Ga, and Al, which significantly reduce transparency of incident light^4^. In addition, even though the several experimental studies on those three dopants have been reported, no guidelines exist for improving the a-C films because the relationship between the structure of a-C films and its characteristics remain unclear, which is a limiting factor for its effective use in any device application^11^. To tackle this issue, it is necessary to theoretically study a-C films due to the limitations of experimental observations on the sub-nanometer scale.

In this paper, we examine the energetics, structure, electronic and mechanical properties of the B, N, and Cl doped a-C films using first-principles density functional theory (DFT) calculations. In our simulation, we generated 64 C atoms-containing amorphous carbon structures doped with B, N, and Cl (concentrations of 1.56~7.81 at.%) to focus on studying dopant content effect on the mechanical properties during the device fabrication processes. We also theoretically investigate the effects of doping in a-C films on the diffusion of F atoms during dry etch process, generating important findings in the field of materials science. If one notes the optimized design with the adequate doped characteristics, our results would provide a new straightforward strategy to tailor the a-C films with excellent mechanical and other novel physical and chemical properties.

## Results and Discussion

### Mechanical properties

**Figure 1:**
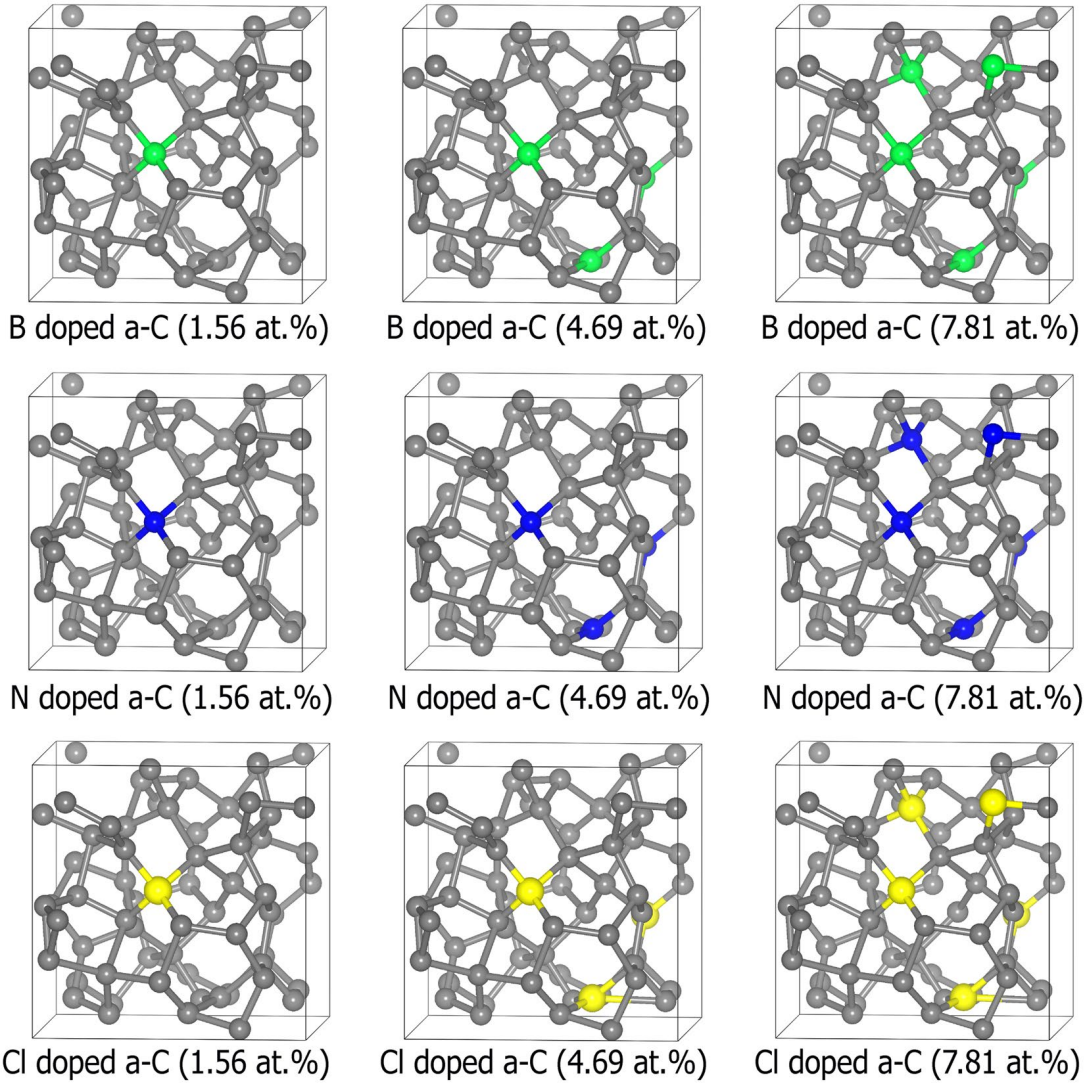
Optimized atomic structures of 64C atoms-containing amorphous carbon structures doped with B, N, and Cl (concentrations of 1.56~7.81 at.%). Grey, green, blue, and yellow colors indicate the C, B, N, and Cl atoms, respectively. Drawings are produced by VESTA^38^ (ver. 3.4.7) software (https://jp-minerals.org/vesta/en/download.html).

Figure 1 shows the final atomic structures of 64C atoms-containing amorphous carbon structures doped with B, N, and Cl (concentrations of 1.56~7.81 at.%). In order to focus on the dopant content effect for the mechanical properties, all the dopant atoms were substituted into the same sites of the amorphous carbon structures. In Table 1, optimized structural parameters (average bond lengths and average bond angles) are estimated from bond length distributions and bond angle distributions in Pure, B, N, and Cl doped a-C films.

**Table 1:** Optimized structural parameters (average bond lengths and average bond angles) estimated from bond length distributions and bond angle distributions in Pure, B, N, and Cl doped a-C films.

	Pure a-C	B doped a-C	N doped a-C	Cl doped a-C
Average bond lengths (Å)	1.522	1.519	1.534	1.584
Average bond angles (°)	111.13	109.46	109.32	104.08

**Figure 2:**
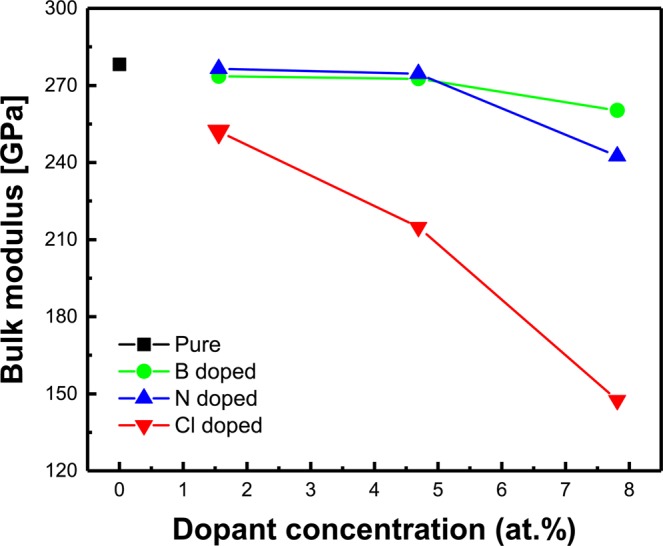
Dependency of the calculated bulk modulus on dopant concentrations for pure, B doped, N doped, and Cl doped a-C films, respectively.

The dependence of the calculated bulk modulus on dopant concentrations is illustrated in Fig. 2. In case of the pure a-C film, the bulk modulus about 278 GPa is estimated. With introducing B and N atoms into a-C films, the bulk modulus as a function of the concentration increases and then are slightly reduced; when the dopant concentration is 7.81 at.%, the minimal bulk modulus of about 260 GPa, 242 Gpa is obtained, which is somewhat reduced by 6~13% compared with the pure a-C. For Cl doped a-C films, the bulk modulus as C1 concentration increases and then reduces; when the Cl concentration is 7.81 at.%, the minimal bulk modulus of about 148 GPa is obtained, which is reduced by 47% compared with the pure case. Similar results have been confirmed by previous experimental results for the film hardness in Cl doped a-C films to investigate friction properties these films using a reciprocating ball-on-disk sliding tester^12^.

Taking the benefits from the simulation, nevertheless, noted that the drastic reduction of bulk modulus is observed by introducing Cl dopant into a-C films, which suggests that the films softened by doping the Cl atom would relieve residual stress of the individual layers within the overall stacks in integrated semiconductor devices. These requirements become more important and increasingly more challenging to meet as the device integrity grows^13,14^.

**Figure 3:**
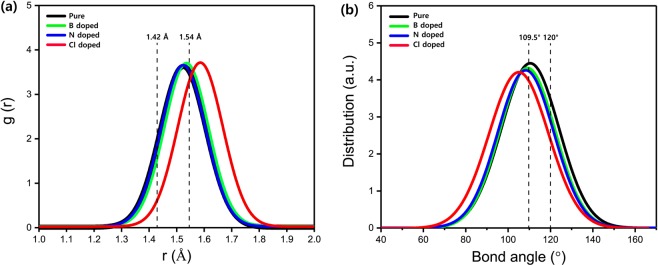
(**a**) Bond length distributions and (**b**) bond angle distributions for pure, B 7.81 at.% doped, N 7.81 at.% doped, and Cl 7.81 at.% doped a-C films, respectively.

In order to clarify the mechanism for giant reduction mechanism of bulk modulus, direct proof was collected first, which is the atomic structure including bond length distributions and bond angle distributions. Calculated bond length and bond angle distributions for the Cl doped a-C film with 7.81 at.% are shown in Fig. 3. For comparison, pure, B doped, and N doped a-C films were also considered. The total bond length and angle distributions in Fig. 3 mainly consists of C−C, C−B, C-N, and C-Cl bond. Li *et al*.^15^ demonstrated that the distortion of both bond lengths (<1.42 Å or >1.54 Å) and bond angles (<109.5° or >120°) in carbon network was an primary factor for the low level of bulk modulus. Therefore, both bond lengths and bond angles in all a-C samples were particularly focused in order to gain the fractions of distorted bonds, which are illustrated Table 2. The black vertical dotted lines in Fig. 3 indicate the equilibrium bond length and bond angle of stable sp^2^ and sp^3^ C−C bonds are 1.42 Å (120°) and 1.54 Å (109.5°) separately.

**Table 2:** Comparison of the fractions of distorted bond length and distorted bond angle estimated from bond length distributions and bond angle distributions in Pure, B, N, and Cl doped a-C films.

	Pure a-C	B doped a-C	N doped a-C	Cl doped a-C
Fraction of distorted bond length (%)	55.4	57.9	55.0	74.3
Fraction of distorted bond angle (%)	68.5	71.4	72.6	79.2

The fractions of distorted bonds in a-C films doped with B, N, and Cl at maximum at.% (7.81%) are thus deduced separately, as shown in Table 2. The calculations for pure a-C film are also carried out for comparison. Figure 3 shows that in pure a-C film the fractions of distorted bond length and bond angle are 55.4% and 68.5%. After monodoping of Cl into a-C film, the fractions are 74.3% and 79.2%, indicating that the drastic reduction of the bulk modulus is attributed to the high fraction of the distorted bonds relaxed by lattice vibrations during stress calculation using VASP. This phenomenon was experimentally confirmed by other researchers^12^. They reported that the Cl doped a-C films deposited using a plasma-based ion implantation and deposition reduce the film hardness and modulus. However, doping of B and N into a-C films show similar values of both bulk modulus and distorted bonds with pure a-C film. Even though the bulk modulus of N-doped a-C is lower than that of B-doped a-C, the fraction of distorted bond length of N-doped is also at a lower value, which is the opposite result. This phenomenon can be explained by the higher fraction of distorted bond angle for N-doped than B-doped a-C films, which is higher impact on reduction of bulk modulus because the distorted bond angle is relaxed larger than bond length by lattice vibrations during stress calculation using VASP. Similar results have been reported by other researchers^4,16^.

### Electronic and structural properties

**Figure 4:**
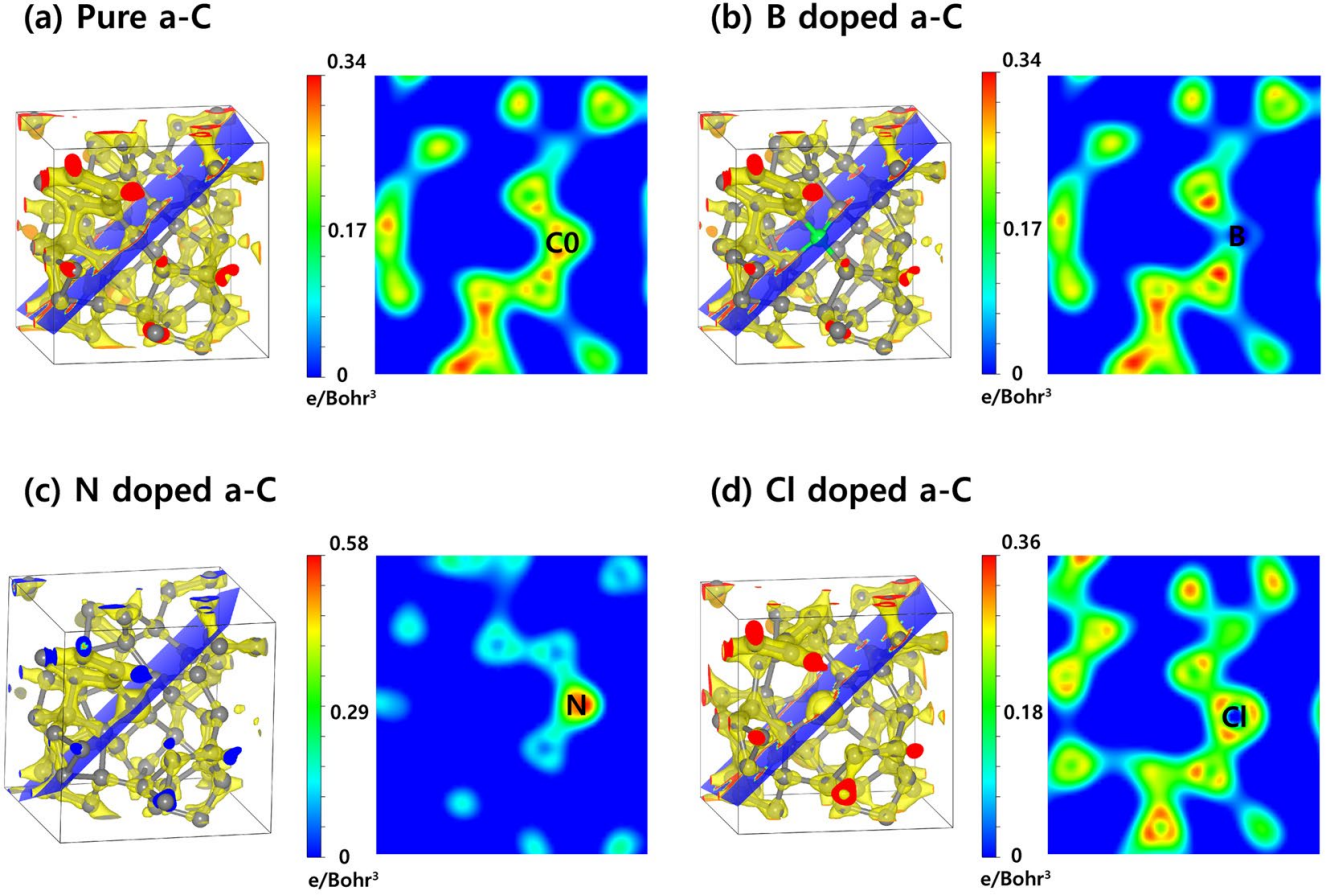
The optimized structures of 3D electron density map (left) and 2D electron density map (right) at 0.025 Å^−3^ iso-surface for the optimized structures of the a-C films; (**a**) Pure, (**b**) B doped, (**c**) N doped, and (**d**) Cl doped a-C films. Drawings are produced by VESTA^38^ (ver. 3.4.7) software (https://jp-minerals.org/vesta/en/download.html).

Figure 4 shows 3D electron density map (left) and 2D electron density map (right) at 0.025 Å^−3^ iso-surface for the optimized structures of the a-C films; (a) pure, (b) B doped, (c) N doped, and (d) Cl doped a-C films. The charge density around boron dopant is clearly lower than surrounding carbons because boron has stronger electron-donation nature than carbon, caused by electronegativity of them (B = 2.04, C = 2.55). However, the value of charge density near nitrogen atom is significantly higher than surrounding carbons since nitrogen has stronger electron-withdrawing nature than carbon, originated from electronegativity of them (N = 3.04, C = 2.55)^17^. For Cl dopant, it has a large electron distribution, suggesting that it has large electrostatic interaction volume even though the electron density is lower than that of N dopant.

**Figure 5:**
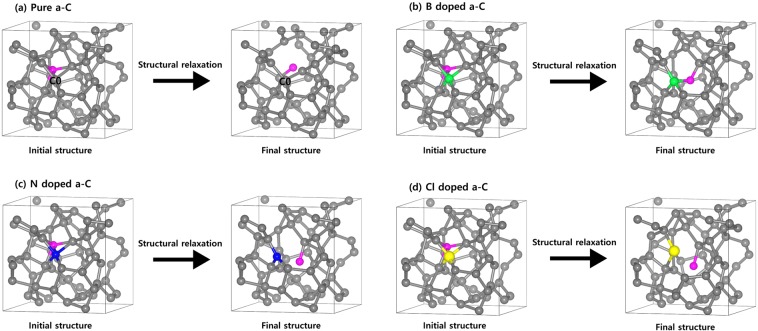
Initial and final structures after structural relaxation of the a-C films when adding an F atom; (**a**) Pure, (**b**) B doped, (**c**) N doped, and (**d**) Cl doped a-C film. Drawings are produced by VESTA^38^ (ver. 3.4.7) software (https://jp-minerals.org/vesta/en/download.html).

Figure 5 shows initial and final structures of the F added a-C films; (a) Pure, (b) B doped, (c) N doped, and (d) Cl doped a-C films. In the final structure of pure a-C films, F atom is captured in sp^2^ carbon near the C0 atom after structural relaxation. The sp^2^ carbon with graphitic structure turns into sp^3^ carbon with tetrahedral structure caused by bonding the F atom. However, in the final structure of B doped a-C, F atom is trapped to B atom, not to the surrounding carbons. This phenomenon that forms stronger B-F bond than C-F bond is attributed to electrostatically strong attractive force between them to strengthen the B‒F bond.

Interestingly, F atom can be bound to the surrounding carbons, but not to the N or Cl atoms due to the energetically unstable bonds to form either N‒F or Cl-F. This can be explained in two perspectives, i.e., electron-withdrawing nature and binding energy. At first, weak bonds of both N‒F and Cl-F would be originated from even high electronegativity of them (N = 3.04, Cl = 3.16, F = 3.98)^17^. This causes electrostatically repulsive force between them to push out. This repulsive force was found when both atoms get close to the F atom during the structural relaxation in our DFT calculation. At second, the weak bonds is attributed to lower binding energy (N-F bond: 3.09 eV and Cl-F bond: 2.60 eV) than C‒F binding energy (5.51 eV), which are well-known values^18^.

### Analysis of doping effect on B, N, and Cl elements

During the dry etch process, injection of etchant molecules such as CF_x_ species leads to the penetration of F atoms through the a-C film up to sub-layer materials. To enhance the performance of the a-C film as an etch hard mask, the a-C film has to block the F atoms on top-surface from diffusion downward up-to the sub-layers, which are very important materials in highly integrated devices. We determined the intermediated states (=energetically stable states) by searching the most stable states between initial and final states after structural relaxation. Even though, in general, complicated molecular reactions have one or more intermediated states (=energetically stable states) between initial state and final state, our calculated results show that the F diffusion from initial state (=top layer) to final state (=bottom layer) has only one intermediated state.

**Figure 6:**
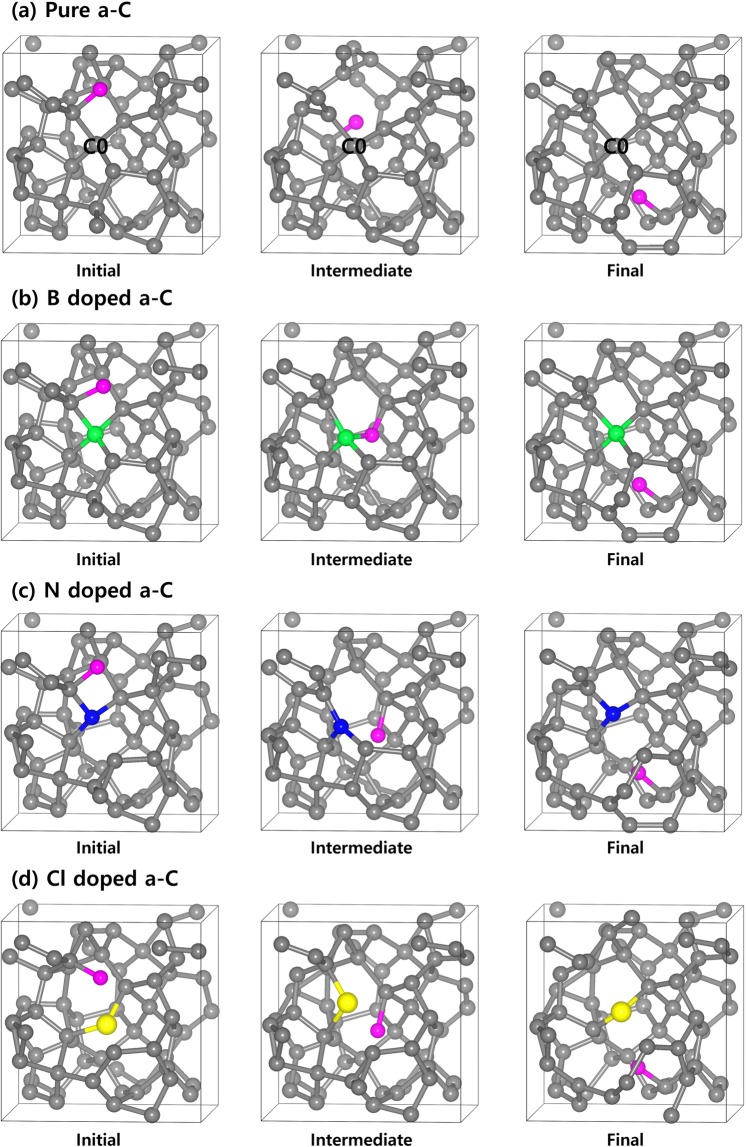
The optimized structures of the initial, intermediate, and final states for the F diffusion in the a-C films; (**a**) Pure, (**b**) B doped, (**c**) N doped, and (**d**) Cl doped a-C films. Drawings are produced by VESTA^38^ (ver. 3.4.7) software (https://jp-minerals.org/vesta/en/download.html).

**Figure 7:**
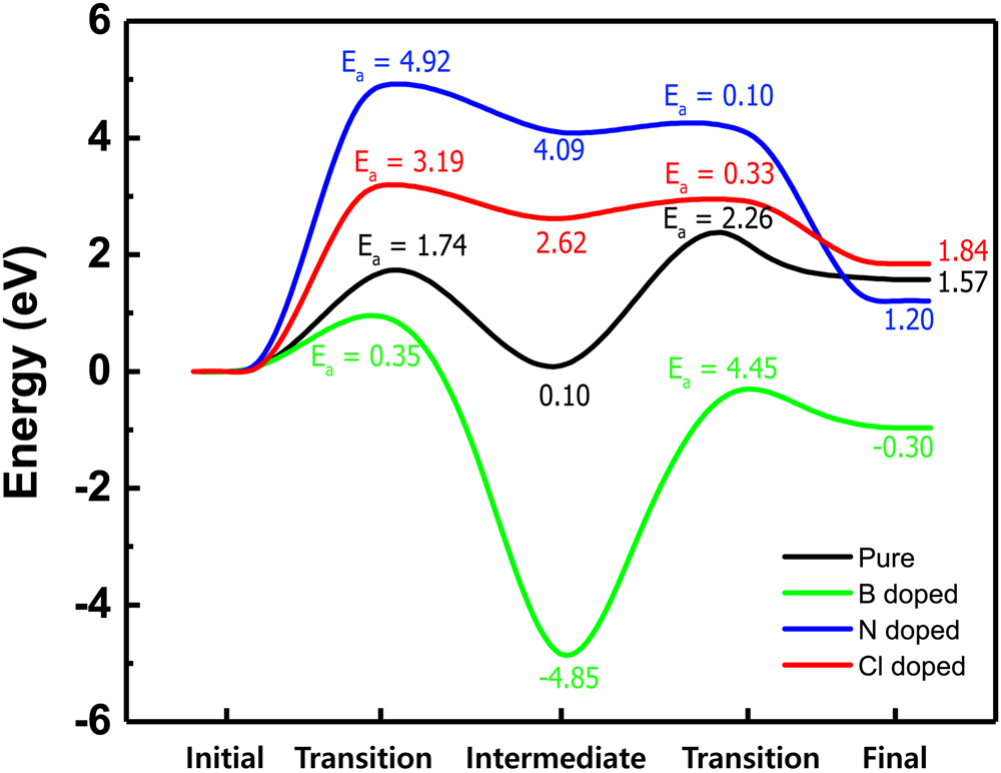
Minimum energy path of F diffusion, corresponding to the initial, transition, intermediate, transition and final states; Pure (black), B doped (green), N doped (blue), and Cl doped (red) a-C films.

For F diffusion to proceed, the F atom can pass by the dopant atom in the a-C films. The optimized structures of the first, second, and third states for the F diffusion in the a-C films are shown in Fig. 6; (a) pure a-C film, (b) B doped a-C film, (c) N doped a-C film, and (d) Cl doped a-C film. Figure 7 shows minimum energy path of F diffusion, corresponding to the initial, intermediate, and final states in Fig. 6. The first diffusion state shows the energy profile of the downward diffusion of F atoms initially adsorbed on the top-layer of the a-C films. In Fig. 6(a), pure a-C film shows F atom diffuses downward and is bound to sp^2^ carbon atom near C0. Although intermediated state can have either lower or higher energy than final state, meta-stable state, which belongs to intermediated state^19,20^, has always higher energy than final state. However, both “intermediated state” and “meta-stable state” terms have one thing in common with lower energy state than transition state. As shown in Fig. 7, energy paths of F diffusion for the four samples of a-C films have each intermediated state. Among the four intermediated states, there are two meta-stable states, which correspond to the intermediated states of N doped (blue line) and Cl doped (red line) a-C films because they have higher energy of intermediated states than their final states.

The interesting point was found that the F atom was bound to sp^2^ C atom by moving somewhat long distance passing by the surrounding sp^3^ C atoms during the structure relaxation. This implies that sp^2^ C atoms play an important role in determining the diffusion path of F atom because of the higher reactive nature of sp^2^ C than sp^3^ C^11^. The diffusion of F atom has two diffusion steps with energy barriers of 1.74 eV and 2.26 eV as shown in Fig. 7. For the B doped a-C film as shown in Fig. 6(b), F atom diffuses downward and is bound to the B atom. Unlike the pure a-C film, F atom is strongly captured by the B atom as it shows small activation energy of 0.35 eV for F diffusion into the B atom (Initial → Intermediate) but, large activation energy of 4.45 eV for F diffusion out of the B atom (Intermediate → Final). Aforementioned in the section 3.2, this phenomenon comes from the strong bonding nature of B‒F bond than C-F bond. For the N doped a-C film, during passing by the N atom (Initial → Intermediate), F atom has extremely large diffusion barrier of 4.92 eV. This large diffusion barrier is attributed to the electrostatically repulsive force between both atoms.

The Cl doped a-C film shows consistently the similar results with the N doped a-C film because both N and Cl atoms have large electro-negativity, which causes F atom to push out of the both atoms. The different point between the N doped and Cl doped a-C films is the value of diffusion barrier (N doped a-C: 4.92 eV, Cl doped a-C: 3.19 eV). This phenomenon can be explained by our DFT calculated electron density map as shown in Fig. 4 because the N doped a-C film clearly demonstrates stronger electron withdrawing nature than the Cl doped one. We need to compare not only activation energy but also energy difference (∆ E = E_after diffusion_ − E_before diffusion_) since the activation energy gives kinetic information and the energy difference gives thermodynamic one. The reason why the kinetic information obtained from activation energy can be very important is because it is possible to estimate how fast diffusion can occur. Thermodynamic information obtained from the energy difference can also be extremely important to check if this diffusion is the endothermic or exothermic reactions. For the endothermic reaction, external energy supply (temperature, light, plasma, etc.) should be accompanied, and for the exothermic reaction, a chain reaction can occur by releasing energy by itself ^21–23^.

Table 3 shows the comparison of the activation energies (E_a_, eV) and energy difference (∆ E = E_after diffusion_ − E_before diffusion_, eV) for F diffusion steps on Pure, B doped, N doped, and Cl doped a-C films. This table clearly shows that doping effect of both N and Cl dopants is to make the diffusion barrier of the F atom be even higher and suppress the F diffusion, meaning that the F atom would not follow the diffusion path passing by the both dopants due to the significant high barriers of 4.92 eV and 3.19 eV, respectively.

**Table 3:** Comparison of the activation energies (E_a_, eV) and energy difference (∆ E = E_after diffusion_ − E_before diffusion_, eV) for F diffusion steps on pure, B doped, N doped, and Cl doped a-C films.

Surfaces	Initial state → Intermediate state		Intermediate state → Final state	
	E_a_	∆ E	E_a_	∆ E
Pure a-C	1.74	0.10	2.26	1.47
B doped a-C	0.35	−4.85	4.45	4.55
N doped a-C	4.92	4.09	0.10	−2.89
Cl doped a-C	3.19	2.62	0.33	−0.78

Even though Cl doped a-C film has not been reported for etch hard mask, our DFT calculated results suggest that Cl doped a-C film would have outstanding characteristics such as mechanical property and blocking nature of F atom for etch hard mask in the ultra-high integrated semiconductor devices. In our DFT calculated results, the combinational doping of both N and Cl into a-C films would provide deep insight into improving the mechanical properties and etch selectivity of the films, leading to enhancing the performance of the future memory devices.

## Conclusions

To summarize, we have demonstrated that B and N dopants inside a-C films makes the bulk modulus somewhat reduced as the concentration increases. We found that, after doping Cl atoms into a-C films, the bulk modulus significantly reduced, which implies that the films softened by doping Cl atoms can help to relieve residual stress for the overall stacks in integrated memory devices. As for the penetration of F atom, B dopant pulls in and captures the F atom inside a-C films, which is attributed to the strong bonding nature of B‒F than C-F bond. However, for the N dopant, F atom has extremely large diffusion barrier of 4.92 eV when passing by the N atom in a-C films. This large energy barrier stemmed from the electrostatically repulsive force between both atoms. Finally, the Cl dopant in a-C films provides consistently the similar results with the N dopant because both atoms have large electro-negativity, which makes F atom to push out. In order to adjust the a-C films with excellent mechanical, physical and chemical properties, our DFT results would give a new straightforward strategy for the optimized design with the adequate doped properties.

## Methods

In our theoretical results, all DFT calculations were performed using Vienna ab initio simulation package (VASP) program with the Perdew-Burke-Ernzerhof (PBE) functional in the generalized gradient approximation (GGA)^24,25^. We used PBE-D2 functional^26^ based on projector augmented wave (PAW) method^27^ with a correction to the conventional Kohn-Sham DFT energy to treat the van der waals interactions for all DFT calculations.

Ab initio molecular dynamics (AIMD) simulations were carried out to obtain the amorphous carbon structures. The cubic supercell of 64 atoms with the fixed lattice constant of 7.61 Å was used, which corresponds to the density of the amorphous carbon with 2.9 g/cm^3^. The melt-quench simulations^28,29^ were performed by pre-melting for 2 ps at 12000 K, melting for 10 ps at 5000 K, and quenching to 0 K with a constant cooling rate of −250 K/ps. For designing the doped amorphous structures, the dopant concentrations for B, N, and Cl were selected ranging from 1.56 to 7.81 at.%, corresponding to 1, 3, and 5 atoms in 64-atom models, respectively. Finally, the amorphous structure was comprised of tetra-coordinated C fraction of 56.3%. Our calculated structures with the coordinated percentage and the density are in good agreement with other theoretical calculations^30–33^. Distances between nearest neighbors for all amorphous samples were determined using a sphere with a cut-off radius of 1.8 Å, obtained by calculating the first minimum value in radial distribution function (RDF). The detailed methodology used to compute the elastic constants of amorphous films was explained in the Supporting Information.

Interactions between ions and electrons were described using Ultrasoft Vanderbilt-type pseudopotentials^34^ with a plane-wave basis set with the cutoff energy of 400 eV. For all amorphous structures, the Brillouin zone was sampled with a 3 × 3 × 3 Monkhorst-Pack k-point mesh. For geometry optimization, forces of all atoms minimize to less than 0.02 eV/Å during self-consistent iterations. We determined the energy barriers (activation energies) through the climbing-image nudged elastic band (CINEB) method^35^ using a force tolerance of 0.02 eV Å. For accurate calculation of barriers, CINEB method with more precise algorithm of transition-state search than the NEB method was used^36,37^. This method is made to search one of various states near the transition-state along the reaction path, converging on the highest saddle point.

## Supplementary Material

Supplementary info
