# Supplementary material for: First principles investigation on energetics, structure, and mechanical properties of amorphous carbon films doped with B, N, and Cl
Source: Sci Rep. 2019 Dec 12;9:18961. doi: 10.1038/s41598-019-55488-x (PMC6908623; doi:10.1038/s41598-019-55488-x)
Supplement: Supplementary file 1 — Supplementary info [file 41598_2019_55488_MOESM1_ESM.docx]

**Electronic Supplementary Information**

First principles investigation on energetics, structure, and mechanical properties of amorphous carbon films doped with B, N, and Cl

Hwanyeol Park,^1,2^ Daekwang Woo,^2^ Jong Myeong Lee,^2^ Se Jun Park,^2^ Sungwoo Lee,^1^ Ho Jun Kim,^3^ Euijoon Yoon^*,1, 4^ and Gun-Do Lee^*,1, 4^

^1^Department of Materials Science and Engineering, Seoul National University, Seoul 08826, Korea

^2^Memory Thin Film Technology Team, Giheung Hwaseong Complex, Samsung Electronics, 445-701, South Korea

^3^Department of Mechanical Engineering, Dong-A University, Busan 49315, South Korea

^4^Research Institute of Advanced Materials and Inter-university Semiconductor Research Cen-ter, Seoul National University, Seoul 08826, South Korea

Corresponding authors: eyoon@snu.ac.kr; [gdlee@snu.ac.kr](mailto:gdlee@snu.ac.kr)

This supporting information explains the detailed methodology used to compute the elastic constants of amorphous films.

In case of crystalline systems, elastic constants can be determined by computing the energies of deformed unit cells. For cubic type phases, distortions with tetragonal and orthorhombic shear, and isotropic distortion along the three lattice vectors require three independent elastic constants C11, C12, and C44 (all elastic constants are expressed using the Voigt notations^1^). For the tetragonal phases, six different deformation modes need to compute C11, C12, C13, C33, C44, and C66. For expansion along three high-symmetry directions, three monoclinic distortions, and three orthorhombic distortions needs to compute nine independent elastic constants.

Next, we discuss the methodology used to compute the elastic constants of amorphous alloys because the amorphous phases are isotropic. Elastic constants, C_ij_ can be obtained by computing the energies of the deformed unit cells; the deformation strain tensor, e_ij_ with six independent components is represented using Voigt notation.

$$e_{ij}=\left( \begin{matrix} e_{1} & e_{6}/2 & e_{5}/2 \\ e_{6}/2 & e_{2} & e_{4}/2 \\ e_{5}/2 & e_{4}/2 & e_{3} \end{matrix} \right)$$

In order to obtain three independent elastic constants C11, C12, and C44 for cubic structure, orthorhombic, isotropic and monoclinic distortions are applied.

The total energy change related to the strain tensor gives

$$E\left( V,e_{ij} \right)= E_{0}+V\sum_{ij} \sigma_{ij}e_{ij}+\frac{V}{2}\sum_{ijkl} C_{ijkl}e_{ij}e_{kl}+O\left[ e_{ij}^{3} \right]$$

where E_0_ and E(e_ij_) are the internal energies of the initial and the strained lattice, respectively; V is the volume of the unstrained lattice; σ_ij_ is the stress; O(e_ij_^3^) indicates the neglected terms in the polynomial expansion. The Elastic stiffness tensors are calculated by the computation of the derivatives of the total energy respect to the applied strain.

$$C_{ijkl}=\frac{1}{V}\left[ \frac{\partial^{2}E\left( V,e \right)}{\partial e_{ij}\partial e_{kl}} \right]_{e=0}$$

Once the stiffness tensor, C_ijkl_, are obtained, elastic properties such as poisson’s ratio, Young’s, bulk, and shear moduli can be calculated by using Voight-Reuss-Hill approximation.^2^

$$K=\left( K_{V}+K_{R} \right)/2$$

$$K_{V}=\frac{1}{9}\left( C_{11}+C_{22}+C_{33} \right)+\frac{2}{9}\left( C_{12}+C_{13}+C_{23} \right)$$

$$K_{R}=\frac{1}{\left( S_{11}+S_{22}+S_{33} \right)+2\left( S_{12}+S_{13}+S_{23} \right)}$$

**References**

1. Leuken, H. v.; de Wijs, G. A.; van der Lugt, W.; Groot, R. A. d., *Physical Review B* **1996,** *53* (16), 10599-10604
2. Chung, D. H.; Buessem, W. R., *Journal of Applied Physics* **1968,** *39* (6), 2777-2782
